# Effects of testosterone and high‐dose anabolic steroids on orthodontic‐induced bone remodeling and root resorption: An animal study

**DOI:** 10.1002/jper.70068

**Published:** 2026-01-26

**Authors:** Caio Luiz Bitencourt Reis, Kelly Galisteu‐Luiz, Gabriela Leite Pedroso, Gustavo Lopes Puls, Leticia Cassaro, Bruno Boaventura Vieira, Fábio Lourenço Romano, Erika Calvano Küchler, Christian Kirschneck, Daniela Silva Barroso de Oliveira, Maria Bernadete Sasso Stuani, Mirian Aiko Nakane Matsumoto

**Affiliations:** ^1^ Department of Pediatric Dentistry School of Dentistry of Ribeirão Preto University of São Paulo Ribeirão Preto São Paulo Brazil; ^2^ School of Dentistry Federal University of Alfenas Minas Gerais Brazil; ^3^ Department of Pediatric Dentistry and Orthodontics Federal University of Rio de Janeiro Rio de Janeiro Brazil; ^4^ Department of Orthodontics University Hospital Bonn Medical Faculty Bonn Germany

**Keywords:** bone, orthodontics, periodontal ligament, root resorption, testosterone

## Abstract

**Background:**

This study investigates the impact of disruptions in testosterone levels on bone remodeling, root resorption, and periodontal ligament (PDL) during orthodontic tooth movement (OTM) in a pubertal male rat model.

**Methods:**

Testosterone deficiency was induced through orchiectomy, and the anabolic‐androgenic steroid (AAS, testosterone undecanoate) was administered in both replacement and high doses. OTM was simulated using a closed‐coil spring on the maxillary right first molar. The surrounding tissues—alveolar bone and periodontal ligament—of both the moved tooth and the contralateral (control) tooth were analyzed 5 and 10 days post‐OTM using micro‐CT, reverse‐transcription quantitative polymerase chain reaction (RT‐qPCR), and immunohistochemistry. Root resorption, testosterone, and adrenocorticotropic hormone plasmatic levels were also evaluated.

**Results:**

Both testosterone deficiency and high‐dose AAS lead to significant changes in bone microarchitecture, resulting in reduced trabecular thickness, decreased bone connectivity, and bone lacunae. Testosterone dysfunction was associated with greater rotation and intrusion of the moved tooth. High‐dose AAS intensified the inflammatory infiltrate and root resorption. Moreover, testosterone dysfunction altered the expression of key genes involved in bone metabolism, including Runx2, Bmp2, Spp1, and Bglap. The Rank/Rankl/Opg pathway was also deregulated due to testosterone disturbances. AAS at replacement doses did not normalize the inflammatory infiltrate, OTM, and the expression of the studied genes to control levels.

**Conclusions:**

Testosterone dysfunction whether from deficiency or high‐dose AAS exposure negatively impacts OTM, increasing bone resorption and promoting inflammation, potentially leading to long‐term consequences for bone health and periodontal support. AAS at replacement doses may also impact PDL and bone during OTM.

## Plain language summary

This study investigated the impact of testosterone and anabolic steroids on tooth movement and the surrounding bone during orthodontic treatment. Using an animal model, we observed how low levels of testosterone and high levels of anabolic steroids impact bone remodeling and root resorption—two key processes involved when teeth are moved with braces. Our findings showed that testosterone and especially high‐dose anabolic steroids significantly altered the typical response of the bone and roots during orthodontic force application. These changes may have implications for the safety and efficiency of orthodontic treatment in individuals with testosterone dysfunction. Understanding these effects is important for both dental professionals and patients, especially given the rising use of anabolic steroids for esthetic or performance reasons. This research helps us better understand how testosterone can influence dental health and may inform more personalized orthodontic treatment plans in the future.

## 1 INTRODUCTION

Orthodontic tooth movement (OTM) is the main way of treating malocclusion. OTM realigns teeth through a process of bone remodeling involving bone resorption and deposition.^1^ Bone remodeling occurs when an orthodontic force is applied to a tooth, triggering reversible molecular and cellular events in the alveolar bone (AB) and the periodontal ligament (PDL). On the compression side (CS), both the AB and PDL undergo compression, leading to hypoxia, vasoconstriction, and inflammation. This cascade of events stimulates osteoblasts and PDL cells to express the receptor activator of nuclear factor κB (RANKL) and various cytokines, which promote osteoclast differentiation and bone resorption. Conversely, on the tension side (TS), the PDL is stretched, triggering the expression of proteins involved in bone deposition, such as osteoprotegerin (OPG), osteopontin (SPP1), osteocalcin (BGLAP), and members of run‐related transcription factor (RUNX), and bone morphogenetic protein (BMP) families.^2^, ^3^ An imbalance in these pathways can result in adverse effects such as severe inflammation, pain, dental root resorption, bone resorption, and loss of periodontal support.^4^ Due to the complex interplay of molecular and cellular events required for healthy OTM and overall bone remodeling, it is essential to explore how both intrinsic and extrinsic factors may influence these processes.

Testosterone, the primary male sexual hormone, plays a crucial role in bone metabolism, especially during puberty.^5^, ^6^, ^7^, ^8^, ^9^, ^10^, ^11^ Testosterone is converted into dihydrotestosterone (DHT), which directly interacts with bone cells through the nuclear androgen receptor (AR). The ligand–receptor complex stimulates the transcription of several proteins involved in bone deposition and resorption, including RANKL, OPG,^8^ BMP2,^12^, ^13^, ^14^ RUNX2,^15^, ^16^ SPP1,^17^, ^18^ and BGLAP.^19^, ^20^, ^21^ The effects of testosterone imbalance on bone health are a growing concern, particularly among adolescents. Testosterone deficiency, or hypogonadism, affects about 5% of male adolescents worldwide.^22^ These individuals often show significant reductions in both bone size and quality compared to their healthy peers. The most common treatment for hypogonadism is testosterone replacement therapy (TRT) using anabolic‐androgenic steroids (AAS).^23^

AAS are synthetic derivatives of testosterone. At replacement doses, AAS can help restore bone growth potential and improve tissue quality in boys diagnosed with hypogonadism.^24^ However, the effects of AAS on muscle building, weight loss, and strength enhancement have led to a surge in their indiscriminate use, particularly among adolescent males.^25^ The lifetime prevalence of AAS abuse has rose significantly in recent years, currently estimated at around 6% among men, reaching 12% among male adolescents.^26^ In Brazil, the Health Regulatory Agency (ANVISA) reported a staggering 670% increase in AAS sales over the past 5 years.^27^ Several AAS users report administering doses 50–100 times higher than physiological levels of testosterone.^28^ As a result, AAS has become a significant substance abuse problem globally in the 21st century, with potentially irreversible consequences.^29^

While the adverse effects of high‐dose AAS on systems such as the cardiovascular, hepatic, and endocrine have been well‐documented,^30^ their impact on bone health remains underexplored.^31^, ^32^ Most research has focused on the therapeutic applications of AAS rather than the high‐dose.^33^ Furthermore, to the best of our knowledge, no study has evaluated the molecular and micro‐morphometric effects of testosterone deficiency and high‐dose AAS in bone remodeling during OTM. Understanding how testosterone imbalance influences OTM has significant clinical relevance: male adolescents frequently undergo orthodontic treatment during puberty, a period marked by hormonal fluctuations, and are also among the main users of AAS for esthetic or performance purposes. These hormonal alterations may affect the rate, stability, and side effects of tooth movement, including root resorption and alveolar bone loss. By experimentally reproducing these conditions, our study provides a translational basis for anticipating and managing orthodontic risks associated with endocrine alterations. Thus, this study aims to simulate testosterone imbalances and OTM in pubertal animal models to investigate the potential adverse effects of testosterone deficiency and high‐dose AAS on bone, PDL, and root.

## 2 MATERIALS AND METHODS

### 2.1 Ethical statement

This study was conducted in accordance with the PREPARE guidelines (Planning Research and Experimental Procedures on Animals: Recommendations for Excellence),^33^ which emphasize careful planning, study design, and quality control in animal research. Additionally, this manuscript was prepared in compliance with the ARRIVE 2.0 guidelines (Animal Research: Reporting of In Vivo Experiments),^34^ which aim to promote transparent and comprehensive reporting of in vivo studies. All procedures were carried out in strict accordance with current ethical standards for animal experimentation, including national and international regulatory guidelines. The Local Ethics Committee for the Use of Animals at the School of Dentistry of Ribeirão Preto, University of São Paulo, approved the study (protocol: 037/2021).

### 2.2 Sample size calculation

Two sample size calculations were conducted to estimate the number of animals required for this study. The first calculation aimed to estimate the number of animals needed for micro‐computed tomography (micro‐CT) and histological analysis, using previous results from Kirschneck et al.^4^ to obtain Cohen's D effect size (1.53). The second calculation focused on estimating the number of animals needed for gene expression analysis, using results from Karakida et al.^31^ to estimate Cohen's D effect size (2.55). Independent comparison tests were performed for both calculations using GPower software Version 3.9.1.7 (Franz Faul, Kiel University, Germany), with an alpha error of 5% and a beta error of 20%. The calculations indicated that four animals per group were required for gene expression analysis and eight animals per group for micro‐CT and histological analysis, resulting in a total of 96 animals for the entire study.

### 2.3 Study design

Male heterogenic Wistar rats were obtained from the Local Bioterium on their weaning day, at an age of 21 days. Three rats were housed per polypropylene cage under controlled temperature (21–23°C) and a 12‐h light–dark cycle, with access to food and water ad libitum. At 23 days old, the animals were randomly allocated into four groups: Sham, orchiectomized group without replacement (ORX), orchiectomized group with testosterone replacement (ORX‐R), and supraphysiological Sham (Sham‐S). The orchiectomy surgery involved the bilateral removal of the testicles and epididymis after ligation of the spermatic cord in ORX and ORX‐R groups. The Sham and Sham‐S groups underwent sham surgery to account for potential confounding factors, with their testicles and epididymis moved and left intact.^5^ The surgeries were performed under intraperitoneal anesthesia (10% ketamine hydrochloride, 55 mg/kg, and 2% xylazine hydrochloride, 10 mg/kg; Syntec do Brazil LTDA, São Paulo, Brazil). After two doses of tramadol hydrochloride 50 mg/mL were administered intramuscularly for analgesia (12.5 mg/kg, 12/12 h; Laboratório Teuto Brasileiro S.A., Anápolis, Goiás, Brazil). Two animals were lost during this phase of the experiment. Animals from different groups were not housed together to prevent abnormal aggressive behavior.^5^

Administration of AAS and placebo (castor oil) began on the day of surgery. The ORX‐R group received AAS at replacement doses (100 mg/kg every 28 days). In contrast, the Sham‐S group received AAS at supraphysiological doses (500 mg/kg every 28 days), as described by Callies et al.^35^

**FIGURE 1:**
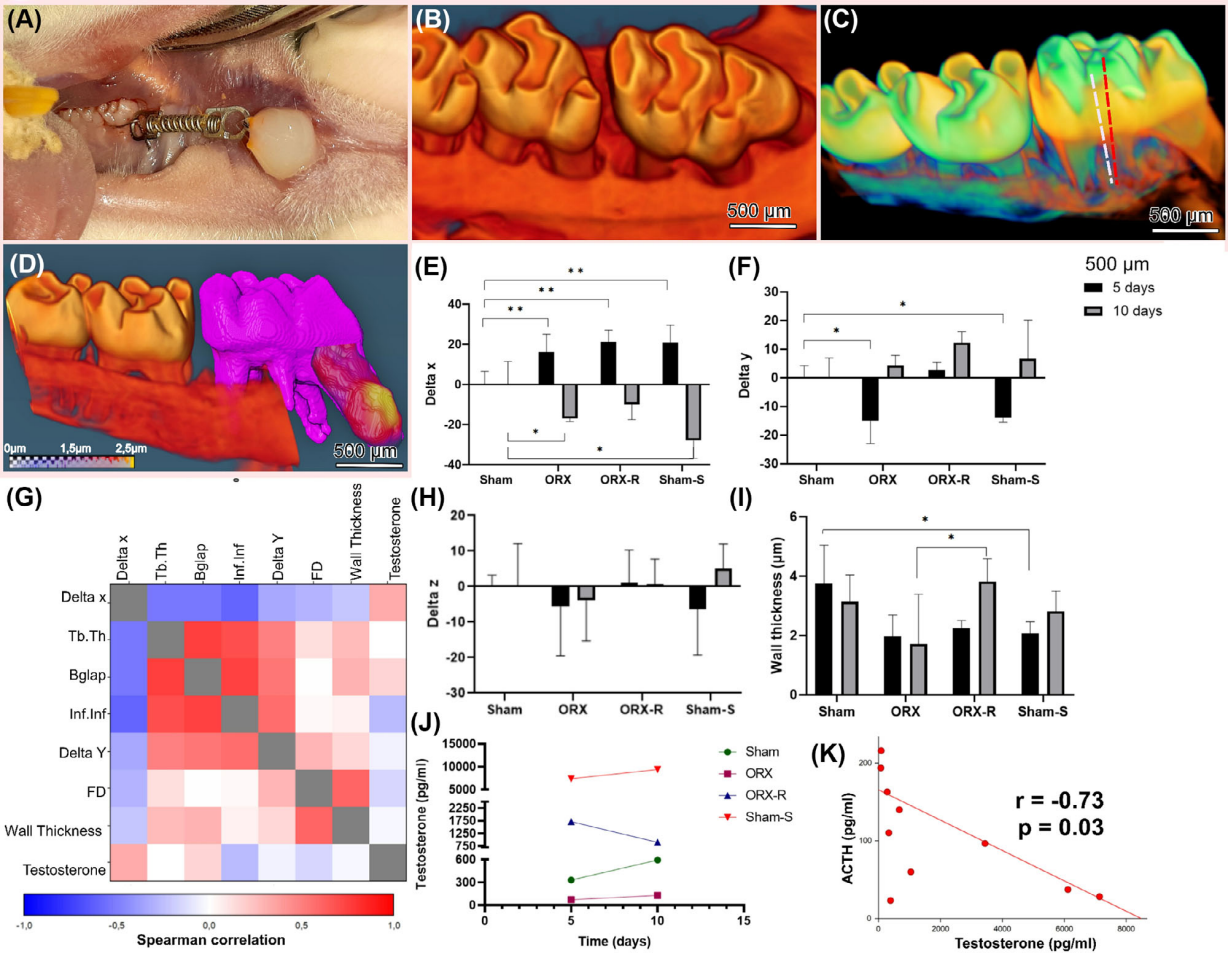
(A) Helicoidal spring distended from the right maxillary first molar to the central incisors on the day of starting orthodontic tooth movement (OTM). (B) Representative 3D reconstruction of the moved side. (C) Superposed 3D reconstructions of the moved side from an animal in the Sham group (orange) and an animal in the orchiectomized group without replacement (ORX) group (blue) to obtain OTM parameters. (D) Representative visualization of the wall thickness method applied to the mesial root of the right maxillary first molar. (E,F,H) Bar graphs comparing delta *x*, *y*, and *z* values, respectively. The delta x estimated the vestibulopalatal rotation of the teeth: values greater than 0 indicated rotation toward the vestibular side, while values less than 0 indicated rotation toward the palatal side, compared to the Sham group. The delta y estimated the horizontal movement of the teeth: values greater than 0 indicated extrusion, and values less than 0 indicated intrusion, compared to the Sham group. The delta z estimated the anteroposterior translational movement of the teeth: values greater than 0 indicated more mesialized, while values less than 0 indicated more distalized, compared to the Sham group. (G) Heat map of the Spearman correlations between some studied variables. (I) Bar graph comparing wall thickness values between groups. (J) Line graph showing testosterone plasma values per group over time. (K) Dot plot graph showing the correlation between testosterone and ACTH plasma levels. * *p* < 0.05; ** *p* < 0.01.

At 50 days old, OTM was simulated on the right maxillary first molar of all animals. A standardized force of 2.5 newtons was applied using a closed helicoidal spring to promote molar mesialization, according to Abi‐Ramia et al.^36^ (Figure 1A). OTM was not simulated on the left maxillary first molar, which served as the control side. After 5 and 10 days of OTM, the animals were euthanized by 100% isoflurane inhalation in a closed chamber. Blood samples were collected after decapitation, and plasma was obtained. Plasmatic testosterone (1:50 dilution) and ACTH (1:5 dilution) levels were measured using enzyme‐linked immunosorbent assay (ELISA; Cayman Chemical Company, Ann Arbor, Michigan, USA) and Milliplex (Merck KGaA, Darmstadt, Germany) tests, respectively, according to the manufacturers.

### 2.4 Micro‐CT analysis

For micro‐CT analysis, the maxilla was fixed in 10% formalin. Both the moved and control sides of each animal were scanned using the SkyScan 1273 micro‐CT system (Bruker Co., Billerica, MA, USA). The scanning parameters for micro‐CT were as follows: 70 kV voltage, 200 µA current, 200 ms time, 1500 images, voxel size 17 µm. Tridimensional images (Figure 1b) were analyzed by Amira 3D Version 2022.2 (ThermoFisher Scientific, Inc., Massachusetts, USA) and ImageJ software (National Institutes of Health, Bethesda, MD, USA).

Bone microarchitecture patterns were assessed from the volumes of interest (VOIs): the alveolar process surrounding the maxillary right (moved) and left (control) first molar. A single and expert operator (C.L.B.R.), whose calibration was validated using the intraclass correlation index (ICC = 0.88), performed the manual segmentation of the VOIs. The criteria to standardize the segmentation limits are detailed in Figure S1 in the online *Journal of Periodontology*. Afterward, morphometric measurements were conducted using the BoneJ plugin in ImageJ software. The Gaussian filter was applied (2.0 on each axis), and the images were binarized, excluding structures (periodontal ligament, background, and potential contaminants) with intensity values less than or equal to 39 on the grayscale (which ranges from 0 to 255). Bone with intensity values of 40 or higher, which correspond to bone tissue with a mineral density exceeding 0.632 g/cm^3^ of hydroxyapatite (Walker et al., 2021), was selected for analysis. After completing these steps, the following bone quality values were obtained: bone volume fraction (mineralized bone volume/total segmentation volume in mm^3^; BV/TV); average thickness of the bone trabeculae (in mm^2^; Tb.Th); density of bone trabecular connectivity (in µg/mm^3^; Conn.D); degree of anisotropy (DA); fractal dimension (FD); number of holes and lacunas (*n*). The purify filter was applied before obtaining connectivity density.

To measure OTM parameters, the moved side 3D reconstruction of each animal from ORX, ORX‐R, and Sham‐S (experimental) groups was superposed on the moved side of each animal from the Sham (control) group, according to Rizk et al.^37^ (Figure 1C). The region of the maxillary right second and third molars was selected as a reference from superposition. To refine the superposition, the Euclidean transformation algorithm was applied. A point was added under the central fossa of the maxillary right first molar, and the Eulerian's coordinates (*x*, *y*, and *z*) were estimated for each animal. A delta value was obtained for each coordinate by the subtraction of experimental group data and control (Sham) results. The delta x estimated the vestibulo–palatal rotation of the teeth: values higher than 0 indicated rotation toward the vestibular, and values lower than 0 indicated rotation toward the palatal, compared to the Sham group. The delta y estimated the horizontal movement of the teeth: values higher than 0 indicated extrusion, and values lower than 0 indicated intrusion, compared to the Sham group. The delta z estimated the anteroposterior movement of the teeth (translational movement); values higher than 0 indicated more mesialization, and values lower than 0 indicated more distalization, compared to the Sham group.

Another operator (L.C.), who had been previously calibrated by the ICC (0.78), manually segmented the mesial root of the maxillary right (moved) and left (control) for root resorption analysis. The entire mesial root was segmented in terms of width and height at the margins of the periodontal ligament and floor of the pulp chamber. The wall thickness method was employed to measure the average cementum thickness between the groups, utilizing Amira 3D software, as described by Zheng et al.^38^ (Figure 1D). The segmentations were binarized to exclude only the background while retaining all structures within the grayscale range of 1–255. The average thickness of the cementum was measured in micrometers over the entire length of the segmented root.

### 2.5 Histological analysis

After micro‐CT, the specimens were washed for 4 h and then decalcified in 4.13% ethylenediaminetetraacetic acid (EDTA), pH 7.0–7.4, until they were fully decalcified for histological analysis (approximately 90 days). The control and moved sides were dehydrated with increasing concentrations of ethanol, treated with xylene, and embedded in paraffin. The paraffin blocks were sliced at 5 µm using a microtome (RM2125; Leica Microsystems GmbH, Wetzlar, Germany) in sagittal planes. For standardization, the slices containing the most extended length of the mesial root of maxillary first molars were chosen for analysis. The slices were mounted on slides (Knittel Gläser, Bielefeld, Germany), subjected to diaphanization and dehydration, stained with hematoxylin and eosin (HE), and then immunoassayed for Runx2, Bmp2, Bglap, and Spp1.

Microscopic images were captured using an AxioPhot (Zeiss, Oberkochen, Germany) connected to a UCMOS Series C‐mount camera (ToupTek Photonics Co., Ltd., Hangzhou, Zhejiang, China). The control side and the compression and tension regions of the moved side were examined. The count of positive and/or negative immunoassayed cells was assessed using QuPath V0.5.1 software^39^ under the following parameters: 100 px background radius and 2 px Sigma, based on 3,3'‐diaminobenzidine (DAB) staining intensity. The percentage of all cells showing positivity was calculated and log10 transformed. To infiltrate inflammatory analysis in HE slices, the module Pixel classification was used to identify and distinguish the infiltrates of other tissues (AB, PDL, dentin, cementum, pulp cells, mesenchymal cells, background, and blood vessels)

Immunohistochemistry was conducted to detect Bmp2, Runx2, Spp1, and Bglap expressed in both the control and moved sides. The slides underwent a pressure cooker pretreatment for 15 min in pH 6.0 sodium citrate buffer. Sections were subsequently incubated with primary antibodies against Bmp2 (1:50 dilution; Santa Cruz Biotechnology Cat# sc‐137087), Runx2 (1:50 dilution; Santa Cruz Biotechnology Cat# sc‐390715), Bglap (1:50 dilution; Santa Cruz Biotechnology Cat# sc‐30045), and Spp1 (1:25 dilution; Santa Cruz Biotechnology Cat# sc‐21742) at 4°C for 16 h. The sections were then treated with secondary antibodies, followed by streptavidin‐biotin‐peroxidase (K0690; Universal Dako LSAB+ Kit, Peroxidase), and DAB (Sigma‐Aldrich). The sections were counterstained using Carazzi's hematoxylin; negative control specimens involved substituting the primary antibody with isotype‐specific serum.

### 2.6 mRNA analysis

reverse‐transcription quantitative polymerase chain reaction (RT‐qPCR) was conducted to assess the gene expression of Bmp2, Runx2, Rank, Rankl, and Opg in the alveolar process surrounding the maxillary right (moved) and left (control) first molar. The maxillary first molars were removed, and bone specimens were preserved in RNAlater (Life Technologies Corporation) at ‐80°C until processing. Total RNA was extracted utilizing the mirVana miRNA Isolation kit (Ambion/Life Technologies). Complementary DNA (cDNA) was generated by reverse transcription using a High‐Capacity Kit (Applied Biosystems). RT‐qPCR was performed on a StepOnePlus sequence detection system (Applied Biosystems™) using TaqMan primers and probes (Runx2, Rn01512298‐m1; Bmp2, Rn00567818‐m1; Rank, Rn04340164‐m1; Rankl, Rn00589289‐m1; Opg, Rn005634990‐m1; Thermo Fisher Scientific, USA). Glyceraldehyde‐3‐phosphate dehydrogenase (*GAPDH)* (Rn01462661‐g1) and *ACTB* (Rn01412977‐g1) acted as endogenous controls and were confirmed to be stably expressed. The relative levels of mRNA expression were determined by the 2∆∆ Cycle Threshold (2‐CT) method. Both *GAPDH* and *ACTB* genes were used for sample normalization according to the protocol by Livak and Schmittgen (2001),^40^ to compute the relative quantitation. For normalization, the CT values of both genes were used to calculate a geometric mean, which was then subtracted from the CT values of the target genes in their corresponding samples.

### 2.7 Statistical analysis

The data were compared using the Kruskal–Wallis test with Duncan's post‐hoc test (*α* = 5%). BV/TV and histological analysis were log10 transformed before statistical analysis. The test and graphs were performed by GraphPad Prism version 10.0.0 for Windows (GraphPad Software, Boston, MA, USA).

## 3 RESULTS

### 3.1 Testosterone dysfunction impairs OTM and affects root resorption

Testosterone dysfunctions resulted in greater tooth rotation (*p* < 0.01; Figure 1E). Additionally, testosterone deficiency and high‐dose AAS led to tooth intrusion (*p* < 0.05; Figure 1F). There was no statistically significant difference between groups regarding anteroposterior movement (*p* > 0.05; Figure 1H). High‐dose AAS caused more root resorption after 5 days of OTM (*p* < 0.05; Figure 1I). After 10 days of OTM, the root resorption in the ORX‐R group was less compared to the ORX group. Details about the testosterone and adrenocorticotropic hormone (ACTH) plasma levels are presented in Figure 1J,K.

Teeth rotation showed correlation with plasma testosterone levels (*r *= 0.32; *p* = 0.040), trabecular thickness (Tb.Th; *r *= −0.52; *p* = 0.005), Bglap expression (*r* = −0.53; *p* = 0.003), and inflammatory infiltrate (*r* = −0.60; *p* < 0.001). Tooth intrusion was negatively correlated with Tb.Th (*r* = −0.43; *p* = 0.003), Bglap expression (*r* = ‐0.53; *p* = 0.009), bone fractal dimension (*r* = −0.29; *p* = 0.037), and inflammatory infiltrate (*r* = −0.57; p = 0.004). Root resorption exhibited a negative correlation with bone fractal dimension (*r* = −0.60; *p* = 0.005). More details about these correlations are provided in Figure 1G.

### 3.2 Bone health is negatively impacted by testosterone deficiency and high‐dose AAS

**FIGURE 2:**
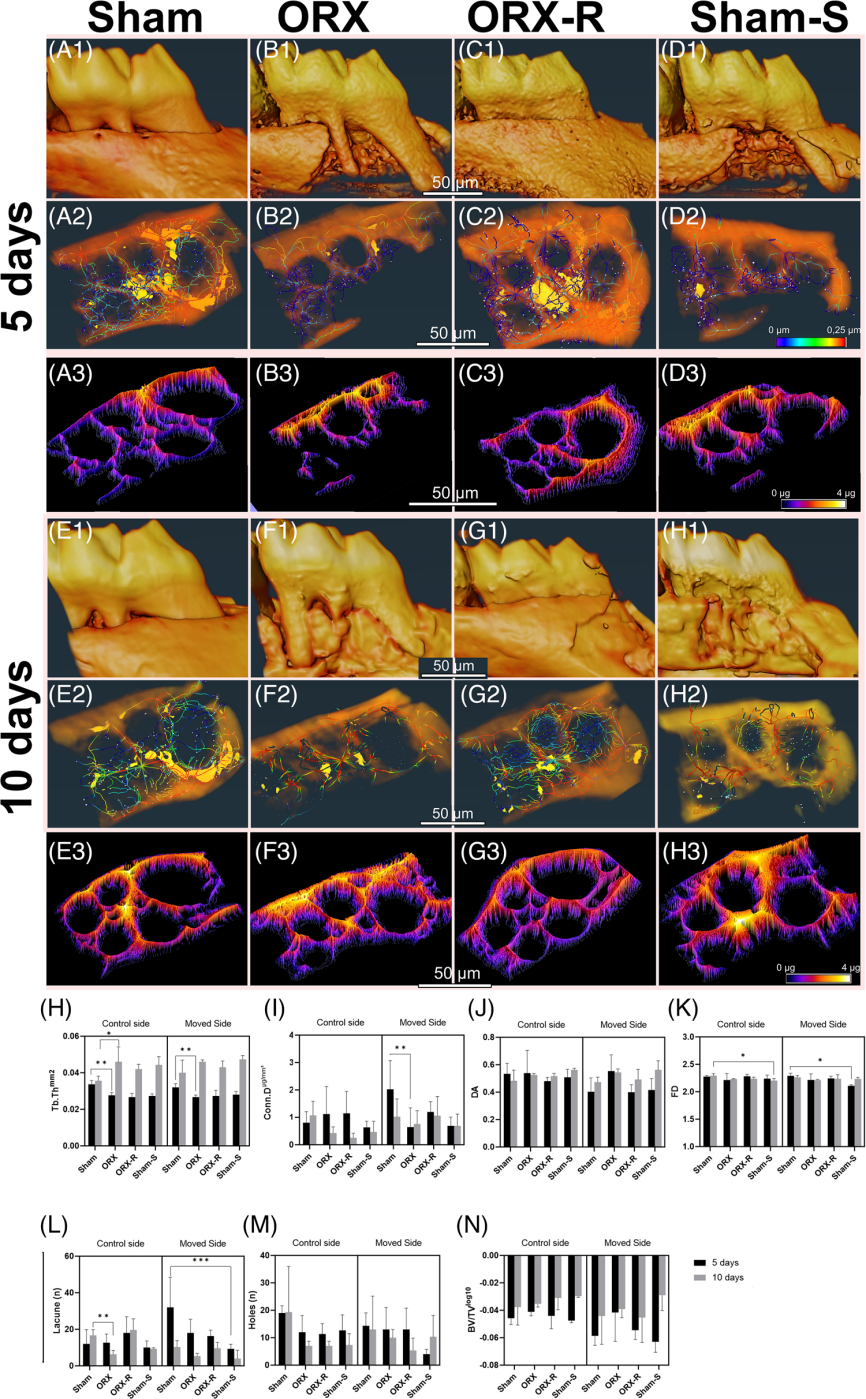
3D reconstructions of the moved side for each group over time. (A1–G1) The raw reconstruction in a sagittal view. (A2–G2) The trabecular distribution and thickness of the alveolar bone (AB), with yellow portions indicating lacunas in a transversal view. (A3–G3) The connectivity density distribution of the trabeculae in the AB in a transversal view. The bar graphs compare the morphometric outcomes for (H) trabecular thickness (Tb.Th mm^2^), (I) connectivity density (Conn.D µg/mm^3^), (J) degree of anisotropy (DA), (K) fractal dimension (FD), (L) number of lacunae, (M) number of holes, and (N) bone volume per total segmented volume fraction (BV/TV log10). * *p* < 0.05; ** *p* < 0.01.

The AB surrounding the right (moved side) and left (control side) maxillary first molars were analyzed using three‐dimensional segmentation to compare the bone microarchitecture between the groups and over time. Figure 2A–G  illustrate the 3D reconstructions of the surrounding AB on the moved side, highlighting aspects such as trabecular distribution, thickness, density, and lacunas.

Testosterone deficiency leads to a decrease in Tb.Th after 5 days of orthodontic tooth movement (OTM) on both the control and moved sides (*p* < 0.001; Figure 2H). However, after 10 days, Tb.Th was found to be greater in the ORX group compared to the Sham group on the control side (*p* < 0.05; Figure 2H). Additionally, testosterone deficiency affects the density of connectivity after 5 days of OTM on the moved side (*p* < 0.01; Figure 2I). The fractal dimension was lower in the Sham‐S group compared to the Sham group on both the control and moved sides (*p* < 0.01; Figure 2K). Furthermore, testosterone deficiency and high‐dose AAS result in fewer lacunas on both the control and moved sides (*p* < 0.001; Figure 2L). The degree of anisotropy (Figure 2J), number of holes (Figure 2M), and bone volume fraction (Figure 2N) did not show statistically significant differences between the groups (*p* > 0.05).

### 3.3 High‐dose AAS increases the inflammatory potential in bone tissue

Histological slices stained with HE (Figure S2a–c in the online *Journal of Periodontology*) were used to evaluate inflammatory infiltrates. High‐dose AAS significantly increased the inflammatory infiltrate on both the compression and tension sides after 5 and 10 days of OTM (*p* < 0.001; Figure S2d). On the tension side, all experimental groups showed a greater inflammatory infiltrate compared to the Sham group after both 5 and 10 days of OTM (*p* < 0.001; Figure S2d). There was no statistically significant difference among the groups on the control side (*p* > 0.05; Figure S2e).

### 3.4 Bone metabolism genes are sensitive to testosterone disturbances

**FIGURE 3:**
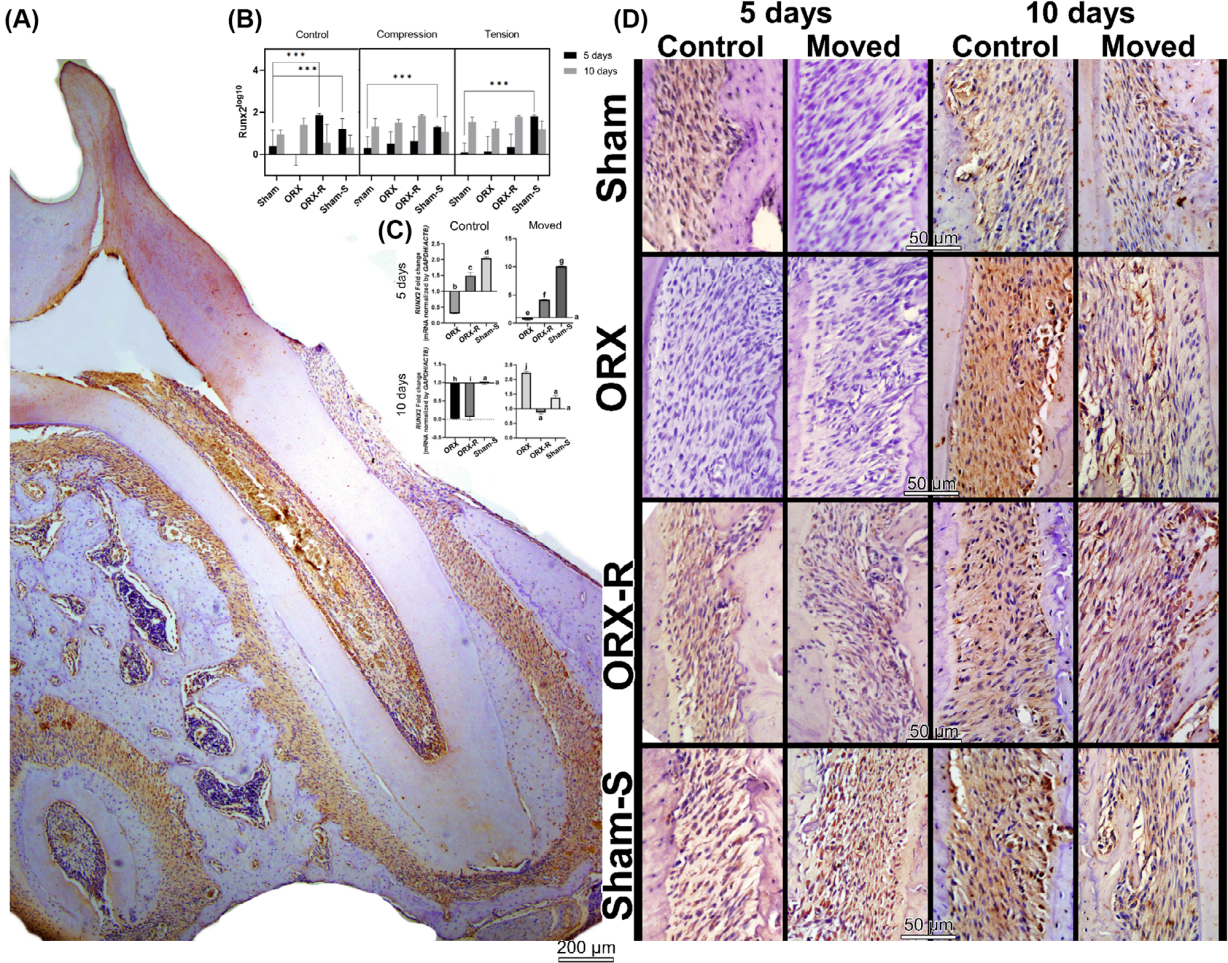
(A) Representative histological slice immunostained for Runx2. (B) Bar graph of Runx2 expression values per group over time for the moved and control sides. (C) Bar graph of mRNA Runx2 values per group over time for the moved and control sides. The additional horizontal axis set at 1.0‐fold change indicate the control group, represented by letter a. The letters over the bars indicate the statistical difference between groups. (D) Representative histological slices per group over time for the moved and control sides. *** *p* < 0.001.

AAS administered at replacement (ORX‐R group) and high doses (Sham‐S group) significantly increases the expression of Runx2 after 5 days on both the control and moved sides (*p* < 0.001). In contrast, testosterone deficiency results in decreased Runx2 expression after 5 days of OTM on both the control and moved sides (Figure 3A–D; *p* < 0.001). After 10 days of OTM, the ORX and ORX‐R groups exhibited lower levels of Runx2 expression on the control side (Figure 3C; *p* < 0.001). However, on the moved side, the ORX group showed an increase in Runx2 expression (Figure 3C; *p* < 0.001).

**FIGURE 4:**
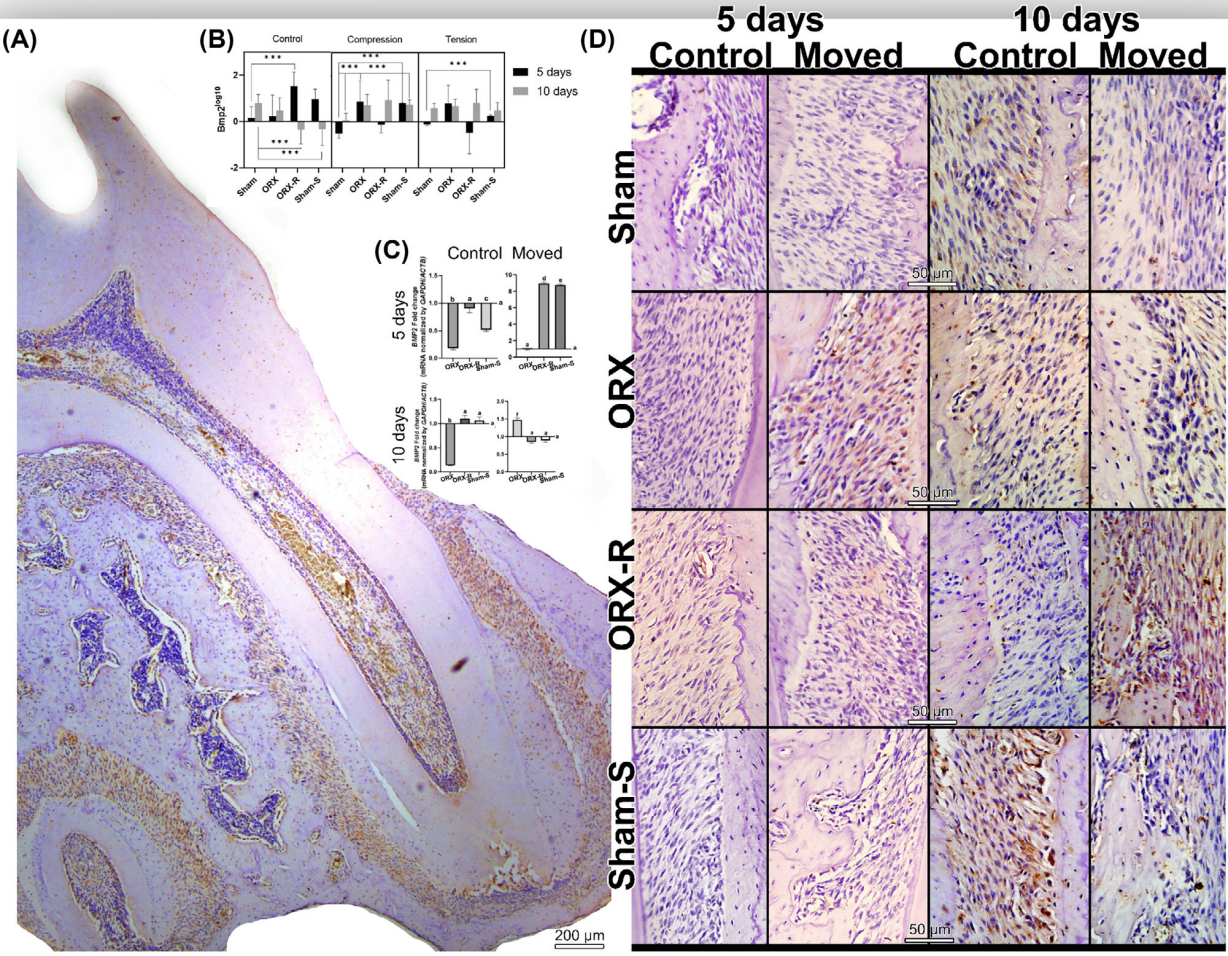
(A) Representative histological slice immunostained for Bmp2. (B) Bar graph of Bmp2 expression values per group over time for the moved and control sides. (C) Bar graph of mRNA Bmp2 values per group over time for the moved and control sides. The additional horizontal axis set at 1.0 fold change indicate the control group, represented by letter a. The letters over the bars (a–j) indicate statistically significant differences between groups; groups sharing different letters are statistically different from each other (*p* < 0.05). (D) Representative histological slices per group over time for the moved and control sides. *** *p* < 0.001.

Bmp2 expression was regulated differently by disturbances in testosterone levels (see Figure 4A–D). In immunohistochemical analysis, the ORX‐R group exhibited higher Bmp2 expression levels on the control side after 5 days of OTM (Figure 4B; *p *< 0.001). After 10 days, both the experimental group and the Sham‐S group exhibited lower Bmp2 expression levels (Figure 4B; *p* < 0.001). On the compression side, the Sham‐S group had higher Bmp2 expression after both 5 and 10 days of OTM (*p* < 0.001), and on the tension side after 5 days of OTM (Figure 4B; *p* < 0.001). In mRNA analysis, testosterone deficiency reduced Bmp2 expression on the control side after 5 and 10 days of OTM. Conversely, on the moved side, testosterone deficiency increased Bmp2 expression. AAS administered at high doses decreased Bmp2 expression on the control side but increased it on the moved side after 10 days of OTM, like the effects seen with AAS at replacement doses. After 10 days, the ORX‐R and Sham‐S groups exhibited Bmp2 mRNA levels similar to those of the Sham (control) group on both the control and moved sides (*p* > 0.05).

**FIGURE 5:**
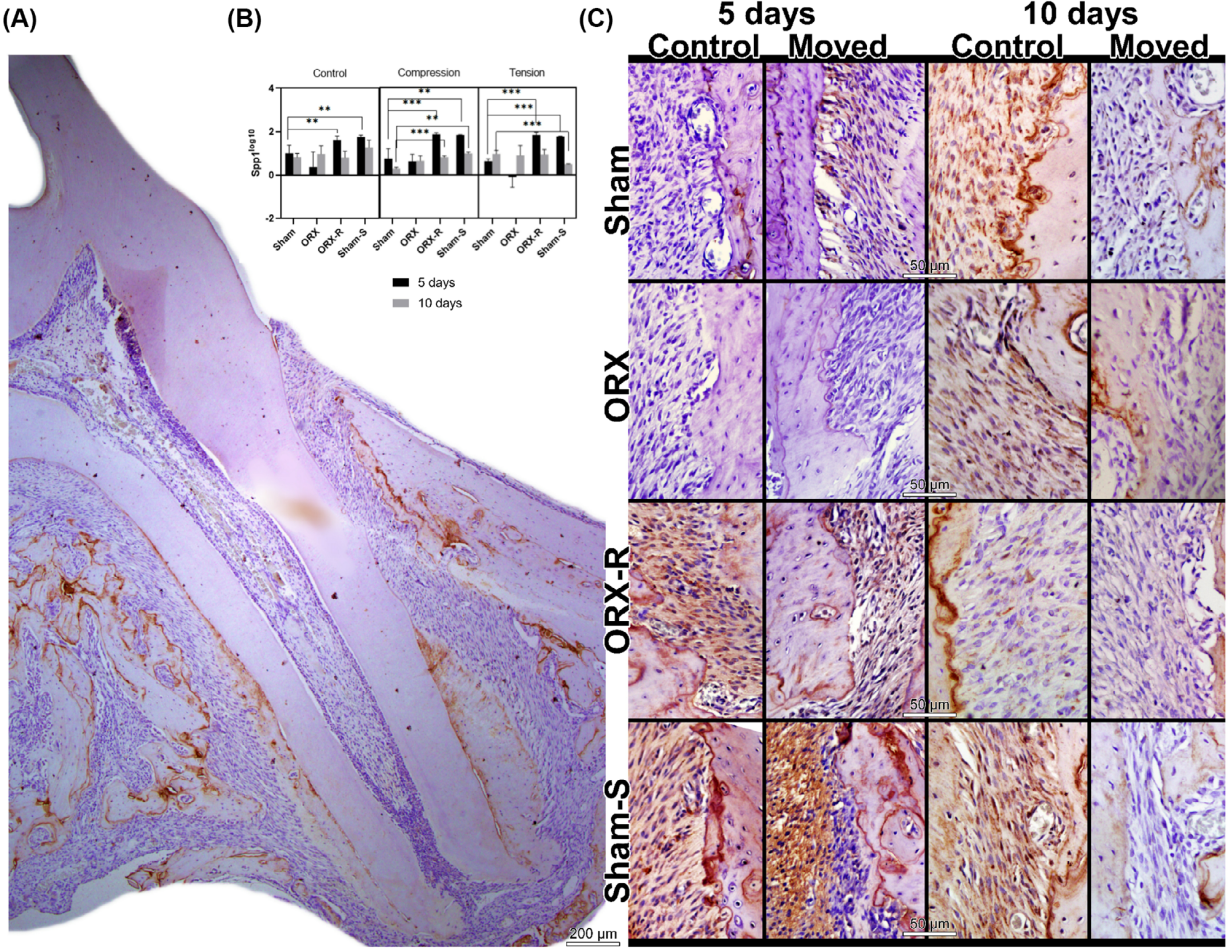
(A) Representative histological slice immunostained for Spp1. (B) Bar graph of Spp1 expression values per group over time for the moved and control sides. (C) Representative histological slices per group over time for the moved and control sides. **p < 0.01; ***p < 0.001.

**FIGURE 6:**
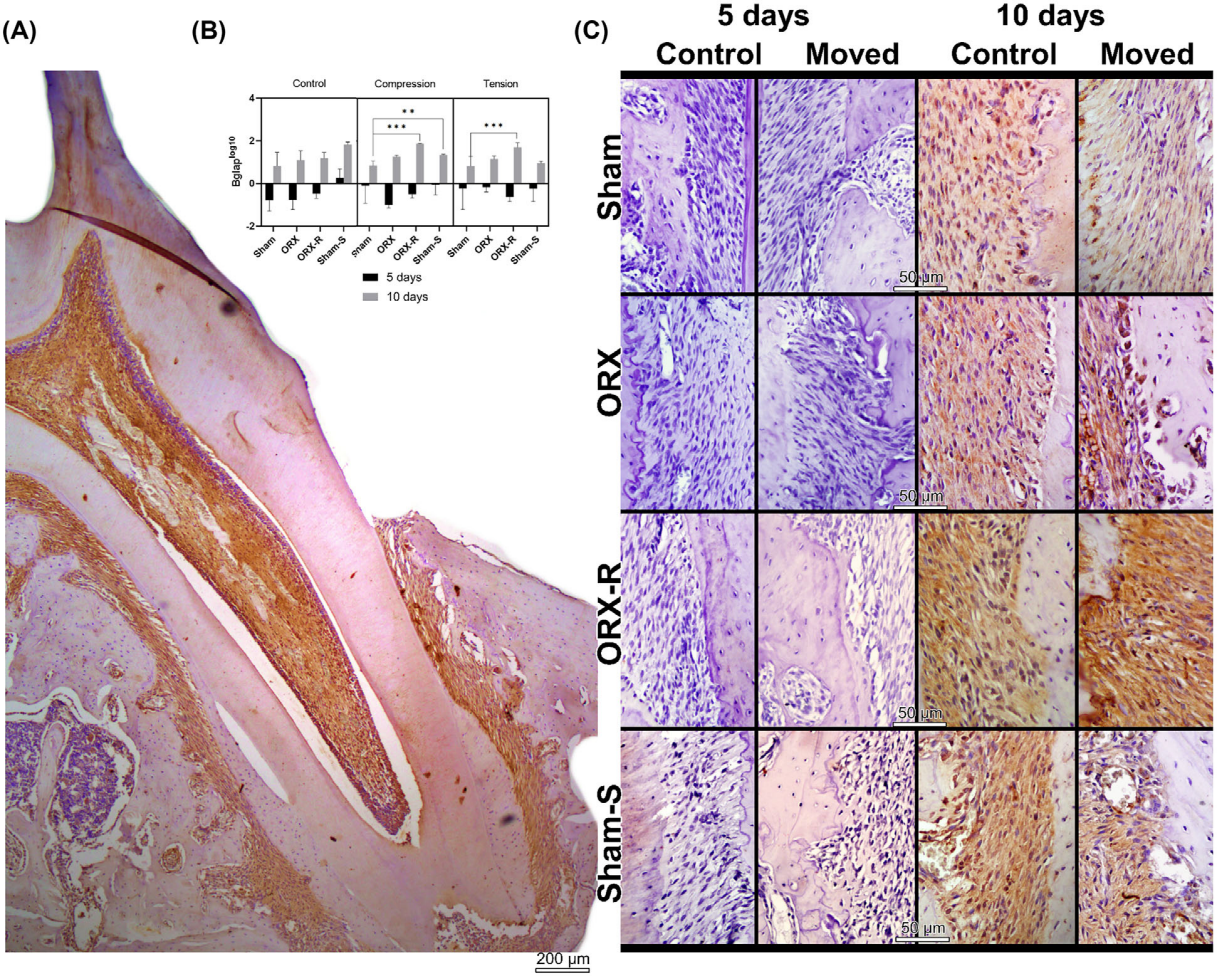
(A) Representative histological slice immunostained for Bglap. (B) Bar graph of Bglap expression values per group over time for the moved and control sides. (C) Representative histological slices per group over time for the moved and control sides. ** *p* < 0.01; *** *p* < 0.001.

AAS at both replacement and high doses increased Spp1 expression on both the control and moved sides after 5 days of OTM (Figure 5A–C). After 10 days, Spp1 expression was higher in the ORX‐R and Sham‐S groups on the compression side; however, Spp1 expression was lower in the Sham‐S group compared to the Sham group on the tension side. AAS at both doses also elevated Bglap expression levels on both the compression and tension sides after 10 days of OTM (see Figure 6A–C). Testosterone deficiency did not affect Bglap and Spp1 expression levels at any experimental time point or on either side (*p* > 0.005).

The mRNA expression levels of the Rank/Rankl/Opg pathway are illustrated in Figure S3A–C in the online *Journal of Periodontology*. Testosterone deficiency led to decreased mRNA expression of all genes on the control side after both 5 and 10 days of OTM (*p* < 0.001). On the moved side, Rank and Rankl levels were higher in the ORX group compared to the Sham group (see Figure S3a,b; *p* < 0.001). AAS at replacement doses increased the levels of all genes on both the control and moved sides after 5 days of OTM (*p* < 0.001). After 10 days, Rankl mRNA levels were elevated in the ORX‐R group on both sides (Figure S3b; *p* < 0.001), while Rank and Opg levels were significantly lower on the moved side in this group (*p* < 0.001). AAS administered at high doses increased levels of all genes on the moved side (*p* < 0.001) but decreased Rank and Rankl levels on the control side after 5 days of OTM (Figure S3a,b; *p* < 0.001). After 10 days, AAS at high doses increased Rankl levels on both sides (Figure S3b; *p* < 0.001) and decreased Opg levels on the control side (Figure S3c; *p* < 0.001).

## 4 DISCUSSION

This study provided unpublished findings concerning the impact of testosterone disturbances on bone tissue and the PDL at the molecular and micro‐morphometric levels. We demonstrated that AB and PDL cells exhibit a negative response to testosterone disturbances during orthodontic‐induced bone remodeling. Specifically, testosterone deficiency leads to decreased bone deposition and quality, while simultaneously increasing bone resorption and inflammatory response. This chain of events contributed to an increase in tooth rotation and intrusion. In cases of high‐dose AAS exposure, there is an overexpression of all genes related to bone metabolism; however, gene expression related to bone resorption overcomes that of bone deposition. Furthermore, high‐dose AAS also amplifies the inflammatory response in AB during OTM, increasing root resorption. Additionally, high‐dose AAS leads to a reduction in the number of bone lacunae due to accelerated bone maturation, which reduces the potential for bone and PDL development, and results in more porous bone, evidenced by the reduced fractal dimension. When AAS was administered at replacement doses, some mechanisms, such as gene expression and the inflammatory infiltrate, remained significantly different from those in the control group during OTM.

The effects of low testosterone levels on bone in humans are well‐documented in the literature, including decreased growth potential, reduced bone mineral density, decreased osteoblastic activity, and increased bone resorption, resulting in an overall imbalance in bone turnover.^6^, ^7^, ^8^, ^9^ Recent systematic reviews suggest that AAS at replacement doses can improve bone health in men with hypogonadism.^41^, ^42^ Besides that, another systematic review also suggest that AAS did not impair bone metabolism among transgender men during gender‐affirming hormone therapy.^43^, ^44^ However, there is a significant lack of evidence regarding the effects of high‐dose AAS on bone. The hypothesis is that high‐dose AAS increases oxidative stress and inflammation in bone cells, thereby accelerating bone maturation.^45^, ^46^ One interesting recent case report^47^ presents a rare complication of osteitis in a 37‐year‐old man, admitted to the hospital with pain and edema five days after a high‐dose AAS administration. Osteitis is characterized by intense inflammation of bone without a marrow space. Our study aligns with previous results regarding testosterone deficiency and confirms the hypothesis about the high‐dose AAS: both scenarios negatively impact bone metabolism, leading to an increased inflammatory response and bone resorption. Our study yields another unprecedented finding: AAS at replacement doses may not normalize specific mechanisms of bone turnover and also increase the inflammatory responses in a mechanical stress situation (OTM).

According to the American Urological Association,^48^ a single injection every 10 weeks of testosterone undecanoate (TU) 750 mg is enough to promote testosterone replacement effects. The dose of TU adopted in our study was referenced by Callies et al.,^35^ which indicates the optimal dose of TU for simulating testosterone dysfunctions in Wistar rats, facilitating clinical translation in humans. We simulated testosterone replacement therapy by administering 100 mg/kg/28 days to the ORX‐R groups, and 500 mg/kg/28 days to simulate AAS supraphysiological levels frequently reported in abusers. When converted by the body surface area scaling method,^49^ these doses correspond approximately to 10–50 times the human therapeutic testosterone replacement dose, consistent with reported abuse regimens in adolescents and athletes.

We evaluated 3D micro‐CT images to measure OTM parameters, root resorption, and bone microarchitecture. Our findings demonstrate that testosterone deficiency and high‐dose AAS lead to unwanted tooth movements during OTM, such as rotation and intrusion. These effects may arise from various factors; however, excessive bone resorption is linked to these unwanted movements.^50^, ^51^ In fact, our study found a correlation between tooth rotation and intrusion and Tb.Th, inflammatory infiltrate, and the expression of Bglap, an important marker for bone deposition.^52^ In contrast, AAS administered at replacement doses appeared to achieve long‐term results in OTM, similar to those observed in the control animals. The root resorption observed in the ORX‐R groups was less compared to the ORX group after 10 days of OTM, while high doses of AAS were associated with an increase in root resorption. We used the wall thickness method to assess root resorption, which evaluates cementum thickness in all dimensions and can detect even small instances of root resorption.^53^

Testosterone deficiency and high doses of AAS impair AB quality, while AAS at replacement doses seem to protect bone tissues against testosterone deprivation.^54^ Testosterone deficiency caused a reduction in Tb.Th, Conn.D, and the number of lacunae, like the effects observed with high doses of AAS, which also led to a reduction in fractal dimension. These findings highlight the disturbance in the resorptive–protective balance of bone homeostasis caused by testosterone disturbances.^5^, ^31^ These effects are apparently mediated by alterations in the Rank/Rankl/Opg axis, a critical determinant of osteoclastogenesis.^6^, ^8^ High doses of AAS upregulated Rank and Rankl expressions after 5 days of OTM; however, the mRNA expression levels of Rank and Rankl were higher than those of OPG. After 10 days of OTM, high doses of AAS continued to increase the mRNA levels of Rankl, while the OPG mRNA levels diminished. It is known that testosterone modulates the Rank/Rankl/Opg axis by suppressing Rankl expression and enhancing Opg transcription in osteoblasts, thereby inhibiting osteoclast differentiation and maintaining a balance between bone resorption and formation.^6^, ^8^ Despite upregulating osteoblast‐related genes such as Runx2, Bmp2, Spp1, and Bglap, high doses of AAS lead to the overexpression of Rankl, particularly under severe inflammatory conditions.^55^

Testosterone has been linked to increased NF‐κB activation, resulting in enhanced production of pro‐inflammatory cytokines (e.g., tumor necrosis factor alpha [TNF‐α], interleukin‐6 [IL‐6]) and secondary stimulation of Rankl expression.^56^ In our study, all experimental groups exhibited an increase in inflammatory infiltrate, with a more pronounced effect observed after 10 days of OTM, suggesting a prolonged inflammatory response rather than an early transient phase. This persistent inflammation may indicate continuous tissue insult caused by hormonal dysregulation, which could maintain osteoclastic activation over time and amplify local bone resorption. The inflammatory infiltrate was also associated with tooth rotation (toward the palatal) and extrusion, confirming the impact of testosterone disturbances in OTM.

The suppression of Runx2 and Bmp2 expression under testosterone deficiency suggests an impairment in osteoblast differentiation and extracellular matrix deposition, contributing to the overall deterioration of bone microarchitecture.^56^, ^57^, ^58^ The increased Bmp2 expression observed in the moved side of the ORX group after 10 days may indicate a delayed compensatory response to counteract resorption‐driven bone loss.^11^, ^12^, ^13^ However, the AAS high‐dose group (Sham‐S) exhibited a decline in Bmp2 expression on the control side, suggesting that excessive AAS exposure may interfere with osteoblast signaling pathways, potentially through altered androgen receptor signaling or oxidative stress‐related mechanisms.

Our findings highlight the critical role of testosterone in regulating healthy bone remodeling, with significant implications for orthodontic treatment and disorders affecting bone metabolism. The results indicate that orthodontic patients with low testosterone levels or those who misuse AAS may exhibit altered responses to orthodontic forces. This could include changes in tooth movement rate, increased lichen planus (LP) inflammation, and alveolar bone loss. This may also explain the interindividual variations in treatment response commonly observed among male adolescents. Testosterone disturbances compromise bone microarchitecture by altering the molecular pathways of bone remodeling, potentially leading to impaired OTM and increased susceptibility to bone loss. High doses of AAS induce an inflammatory response that may further exacerbate bone resorption despite their transient osteogenic effects. AAS at replacement doses may also increase the inflammatory response and fail to regulate the expression of genes involved in bone metabolism. Although this investigation was performed in an animal model, our findings have direct implications for clinical orthodontics and periodontics. In humans, testosterone dysfunctions, such as hypogonadism or self‐administration of high‐dose AAS, can lead to alveolar bone loss and an increased risk of root resorption and inflammatory periodontal responses. Even patients with a medical indication for the use of AAS at replacement doses may experience some effects during OTM, such as an increased inflammatory response. These insights underscore the importance of hormonal evaluation in patients undergoing orthodontic treatment. Orthodontists and periodontists should be aware of these hormonal influences when planning or monitoring treatment in male adolescents with endocrine disorders or AAS use. Hormonal screening may be considered in cases of delayed or exaggerated orthodontic response, unexplained inflammation, or bone loss. Early identification and collaboration with endocrinologists could improve treatment safety and predictability. Future clinical studies are needed to confirm whether testosterone imbalance alters tooth movement rates or root resorption in humans.

We acknowledge that a limitation of our study is that we did not include female rats in our analysis. Future studies should focus on potential targeted therapeutic interventions, such as selective androgen receptor modulators (SARMs) or osteoprotective agents, to mitigate the detrimental impact of hormonal imbalances on skeletal homeostasis. We also suggest that future studies should investigate the effects of bone remodeling in AAS‐induced hypogonadism. Understanding the precise molecular mechanisms underlying these alterations will be essential for optimizing clinical strategies in orthodontics and bone metabolism disorders. Another limitation is that we did not evaluate other limb bones, which makes it difficult to generalize the results to other skeletal structures.

## AUTHOR CONTRIBUTIONS

*Conceptualization*: Caio Luiz Bitencourt Reis, Mirian Aiko Nakane Matsumoto, Erika Calvano Küchler, Christian Kirschneck, and Daniela Silva Barroso de Oliveira. *Methodology*: Maria Bernadete Sasso Stuani. *Software*: Caio Luiz Bitencourt Reis, Leticia Cassaro, and Kelly Galisteu‐Luiz. *Validation*: Bruno Boaventura Vieira, Fábio Lourenço Romano, Daniela Silva Barroso de Oliveira, and Christian Kirschneck. *Formal analysis*: Caio Luiz Bitencourt Reis and Kelly Galisteu‐Luiz. *Investigation*: Caio Luiz Bitencourt Reis, Kelly Galisteu‐Luiz, Gabriela Leite Pedroso, Gustavo Lopes Puls, Leticia Cassaro, and Maria Bernadete Sasso Stuani. *Resources*: Mirian Aiko Nakane Matsumoto and Maria Bernadete Sasso Stuani. *Data curation*: Caio Luiz Bitencourt Reis and Kelly Galisteu‐Luiz. *Writing—original draft preparation*: Caio Luiz Bitencourt Reis, Erika Calvano Küchler, and Mirian Aiko Nakane Matsumoto. *Writing—review and editing*: All authors. *Visualization*: Mirian Aiko Nakane Matsumoto. *Supervision*: Fábio Lourenço Romano, Maria Bernadete Sasso Stuani, and Mirian Aiko Nakane Matsumoto. *Project administration*: Mirian Aiko Nakane Matsumoto. *Funding acquisition*: Mirian Aiko Nakane Matsumoto.

## CONFLICT OF INTEREST STATEMENT

The authors declare that they have no competing interests.

## DECLARATION OF GENERATIVE AI AND AI‐ASSISTED TECHNOLOGIES IN THE WRITING PROCESS

Nothing to disclose.

## PROTOCOL

The protocol of this study (2021) can be found in this link: https://dmptool.org/plans/62818/export.pdf?export%5Bpub%5D=true&export%5Bquestion_headings%5D=true.

## COPYRIGHTED MATERIALS

No material has been reproduced from another source.

## Supporting information



Supporting Information

Supporting Information

Supporting Information

## Data Availability

The data that support the findings of this study are openly available in Google Drive at https://drive.google.com/drive/folders/1H0JeHD8dmivw9rbsUpSU20FzT‐yjY5_‐?usp=drive_link
